# Point Charges Optimally Placed to Represent the Multipole Expansion of Charge Distributions

**DOI:** 10.1371/journal.pone.0067715

**Published:** 2013-07-04

**Authors:** Ramu Anandakrishnan, Charles Baker, Saeed Izadi, Alexey V. Onufriev

**Affiliations:** 1 Department of Computer Science, Virginia Tech, Blacksburg, Virginia, United States of America; 2 Graduate Program in Biophysics, Harvard University, Cambridge, Massachusetts, United States of America; 3 Department of Engineering Science and Mechanics, Virginia Tech, Blacksburg, Virginia, United States of America; 4 Department of Physics, Virginia Tech, Blacksburg, Virginia, United States of America; University of Calgary, Canada

## Abstract

We propose an approach for approximating electrostatic charge distributions with a small number of point charges to optimally represent the original charge distribution. By construction, the proposed optimal point charge approximation (OPCA) retains many of the useful properties of point multipole expansion, including the same far-field asymptotic behavior of the approximate potential. A general framework for numerically computing OPCA, for any given number of approximating charges, is described. We then derive a 2-charge practical point charge approximation, PPCA, which approximates the 2-charge OPCA via closed form analytical expressions, and test the PPCA on a set of charge distributions relevant to biomolecular modeling. We measure the accuracy of the new approximations as the RMS error in the electrostatic potential relative to that produced by the original charge distribution, at a distance 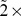 the extent of the charge distribution–the mid-field. The error for the 2-charge PPCA is found to be on average 23% smaller than that of optimally placed point dipole approximation, and comparable to that of the point quadrupole approximation. The standard deviation in RMS error for the 2-charge PPCA is 53% lower than that of the optimal point dipole approximation, and comparable to that of the point quadrupole approximation. We also calculate the 3-charge OPCA for representing the gas phase quantum mechanical charge distribution of a water molecule. The electrostatic potential calculated by the 3-charge OPCA for water, in the mid-field (2.8 Å from the oxygen atom), is on average 33.3% more accurate than the potential due to the point multipole expansion up to the octupole order. Compared to a 3 point charge approximation in which the charges are placed on the atom centers, the 3-charge OPCA is seven times more accurate, by RMS error. The maximum error at the oxygen-Na distance (2.23 Å ) is half that of the point multipole expansion up to the octupole order.

## Introduction

Point multipole expansions are widely used to gain physical insight by providing a simplified expression for a complex distribution of sources of potential fields, such as electrostatic potential due to a charge distribution. Many familiar physics concepts are introduced using the framework of point multipoles because point multipoles provide a means of decoupling the underlying features of a source distribution from the observation point. Furthermore, since each successive term in the multipole expansion decays more rapidly with distance than the previous term, the impact of high order terms becomes small in the far-field, i.e. at distances 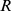 such that 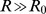, where 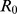 is the distance of the furthest charge from the expansion center. This property has allowed the point multipole expansion to simplify many practical calculations. For example, algorithms such as the fast multipole [1], local reaction field [2], and fitted point multipole (FPM) [3] methods, use point multipoles to reduce the computational complexity of calculating pairwise interactions between large charge distributions. However, at distances not much larger than 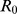, the accuracy of the low order point multipole approximation deteriorates quickly as one approaches the charge distribution, necessitating introduction of higher order terms. This, in turn, may lead to cumbersome algebra and the need to introduce further approximation [4]. Since, in practice, the potential often needs to be calculated in regions where the assumption 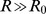 does not hold, the point multipole expansion with only one or two lowest order terms may be suboptimal for some practical calculations. For example, in atomistic molecular simulations, amino acids interacting inside a single protein are often only several (1–5) times 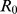 apart. For a typical amino acid group within a folded protein such as lysozyme 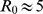 Å, and the distance between amino acid groups ranges from 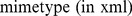 to 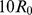. The value of 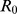 and the distance at which the potential due to the charge distribution is evaluated, is, of course, problem dependent. Furthermore, compared to point charge approximations, it is generally more difficult to implement point multipole approximations into existing molecular modeling software, especially for commonly used implicit solvent models.

Arguably, one of the most successful point multipole based approximations is the fast multipole method [1]. The fast multipole method partitions the system into a hierarchical set of cubic lattices. Electrostatic interactions between charges within a lattice and in neighboring lattices (the near-field) are treated exactly, while a truncated multipole expansion is used for electrostatic interactions due to atoms in the more distant lattices (the mid- and far-field). The size of the lattices used in the multipole expansion varies, with a larger lattice size being used for more distant lattices [5]–[7]. This technique reduces the complexity of the computation of pairwise interaction from 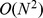 to less than 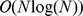, where 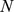 is the number of interacting particles [1]. Many improvements to the original technique have been made [6], [8]–[11]. Overall, the fast multipole method has the advantage of lower computational complexity compared to the full pairwise computation, and has a well defined error bound [1]; the method is used in many areas of physics. However the fast multipole method has not been widely adopted in biomolecular simulations, most likely due to its algorithmic complexity, and the discontinuities in calculated potential inherent in the method. [12], [13] The local reaction field [2] and fitted point multipole (FPM) [3] methods have also not been widely utilized in the context of biomolecular modeling, again most likely due to their algorithmic complexity.

Here we investigate an alternative to the point multipole expansion for approximating charge distributions, which we call optimal point charge approximation (OPCA). Unlike the fast multipole method, which uses a set of point multipoles to represent the original charge distribution, the OPCA approximates a charge distribution using a given number of point charges. These point charges are chosen so that they optimally reproduce the lowest order multipoles in the expansion of the original distribution. Since OPCAs have a finite size, as opposed to being point-like, they may provide better representation of the original spatially extended charge distribution than a single-center truncated point multipole expansion. In particular, a more accurate representation of the potential in the mid-field may be expected. We prove below that the 1-charge and 2-charge OPCAs are at least as accurate as the equivalent order point multipole approximations, i.e. the point monopole and dipole approximations. Throughout this work we refer to point monopole, dipole, and quadrupole approximations as the truncated point multipole expansions upto the monopole, dipole, and quadrupole order, respectively.

We show that in general it is always possible to numerically determine the OPCA, however, for many practical applications, such as molecular dynamics simulations, analytical expressions are needed. Although it is not always possible to derive a practical analytical expressions for OPCAs, in certain cases we show that reasonable, robust and fairly simple approximations to the OPCAs can be derived, which we refer to as the practical point charge approximation (PPCA). The 2-charge OPCA is one such case for which a practical analytical expression is not readily evident for arbitrary charge distributions. For this case we have derived an approximation to the OPCA, the 2-charge PPCA. In what follows we evaluate the accuracy of our approximations for a set of charge distributions relevant to biomolecular modeling at a distance 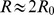. The accuracy at such distances is most critical for multiscale approximations [14], [15] such as the hierarchical charge partitioning method [16]. For smaller distances, multiscale approximations generally use the exact charge distribution in their computations. From a practical standpoint, PPCAs may also be easier than the fast multipole protocol to implement in applications that already utilize point charges, i.e. in many molecular dynamics packages [17], [18].

The rest of this work is organized as follows. We first review the multipole expansion concept to orient the reader and provide a convenient notational reference. Next, we describe the theoretical basis for the optimal point charge approximation. We then use this theoretical formalism to derive closed-form expressions for the optimal and the practical point charge approximations for the 1- and 2-charge cases. The accuracy of the 2-charge PPCA was evaluated for a practical application relevant to biomolecular modeling. We also calculated the 3-charge OPCA for approximating a quantum mechanical charge distribution for a water molecule; the resulting OPCA reproduces the electrostatic potential in the mid-field with greater accuracy than the point octupole expansion. Potential uses and future work are discussed in “Conclusions”.

## Methods

### Multipole Expansion

Here we will give a brief overview of the formalism of the point multipole expansion. Since many practical applications, such as molecular dynamics simulations, use point charges, for notational simplicity we will consider discrete charge distributions, but our main results also hold for continuous distributions.

Consider a set of N point charges 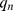 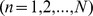 located at positions 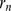 around some chosen origin. Then the potential 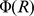, of this distribution at a point 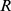 from that origin is given by the familiar Coulomb potential
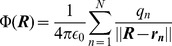
(1)


For distances 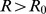 where 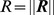 and 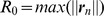, a Taylor series expansion of the potential above gives the classic multipole expansion. In Cartesian coordinates we obtain
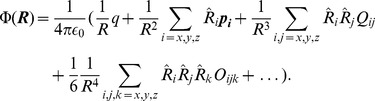
(2)where



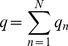
(3)

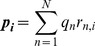
(4)

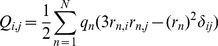
(5)

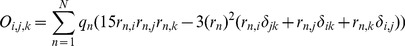
(6)



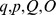
 are known as the monopole, dipole, quadrupole and octupole moments respectively, 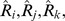 with 
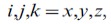
 are the unit vectors along the 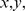 or 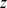 coordinates, and 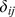 is the Kronecker delta. The multipole moments are symmetric tensors where the lowest order non-vanishing multipole is origin independent.

### The Optimal Point Charge Approximation (OPCA)

For a given set of 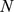 original charges 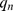 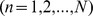, we want to determine the position and magnitude of 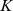 point charges 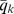 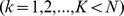 such that the potential due to these smaller number of point charges, 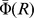 best approximates the potential of the original distribution, 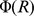. Our criterion for the “best approximation” is as follows: the optimal point charge approximation (OPCA) minimizes the error in the multipole expansion for the 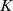 point charges relative to the multipole expansion for the original distribution of 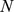 charges. The precise error metric is defined below.

#### The error metric

Determining the best representative charge distribution is contingent upon the definition of the error metric used. In general, we are concerned with obtaining the best representation of the original potential at any arbitrary point in space outside the distribution. Thus, for the error metric, 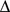, one typically chooses the root mean square (RMS) of the error in potential over some volume 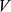 (or surface) excluding the volume 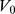 containing the charge distribution being approximated, i.e.
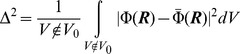
(7)


In principle, one can derive the optimal charge placement 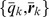 by minimizing the integral given in Eq. (7) with respect to the values of the new charges, 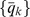 and their positions 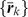. However, as the number of charges in the representative distribution grows, this equation can be expensive to minimize numerically, let alone to find closed-form analytic expressions for the placement and magnitude of the charges composing the representative distribution. In addition, the choice of the integration volume is somewhat arbitrary. Furthermore – and, perhaps, most importantly – charges chosen in this manner are not guaranteed to have the same multipole moments as the original distribution [19]. This can lead to misinterpretation of the properties of the distribution and, potentially, to unphysical results. At the very least, we would like the new approximate representation to inherit the same asymptotic behavior of the corresponding point multipole expansion of the same order, but with greater accuracy expected from a spatially extended distribution that can better mimic the original charge distribution.

To simplify the problem, we recast Eq. (7) in spherical coordinates and consider the error inside a spherical shell centered on the chosen multipole expansion center, and with arbitrary outer radius 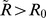, where 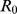 is defined as before, i.e. the distance from the expansion center to the outermost point charge. The error metric now becomes
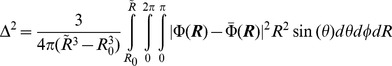
(8)where 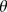 and 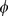 are the usual spherical coordinate inclination and azimuth angles.

In spherical coordinates, the multipole expansion is given by
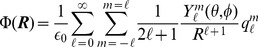
(9)

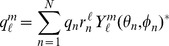
(10)where 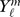 are the standard spherical harmonics, 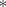 denotes the complex conjugate, 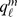 are the spherical multipole moments, and 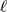 is the multipole order. Using this expansion as our error metric, Eq. (8), becomes
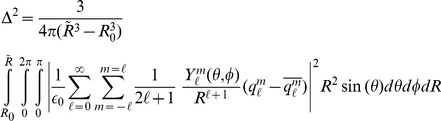
(11)where 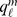 and 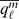 are the spherical moments of the original and representative charge distributions respectively. Since the spherical harmonics are orthonormal, Eq. (11), can be further simplified to the following form [20],
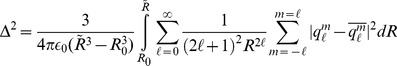
(12)


#### Calculating the optimal point charge approximation

The position and magnitude of the representative point charges in OPCA for a given order 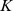 are calculated by sequentially minimizing each term in the error expansion Eq. (12), starting with the lowest order (monopole) term. From the structure of Eq. (12) we see that minimizing the difference between the successive multipole moments of the original 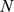-charge and the optimal 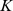-charge distributions is equivalent to minimizing the total error in electrostatic potential. Note that the procedure does not depend on the parameter *R*, and thus the method does not require explicit integration over a given region. This removes a degree of arbitrariness in defining the “error surface” inherent in several other methods currently used in practice. The use of the multipole expansion as reference allows for the sought after distinct separation of terms by the rate at which they decrease as a function of *R*, i.e. the monopole term falls off as 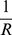, the dipole falls off as 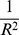, etc. A representation that makes terms up to order 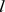 in Eq. (12) equal to zero will produce total error whose leading term falls off as 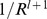. For 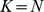, which is the number of charges in the original distribution, the OPCA exactly reproduces the electrostatic potential due to the original distribution. This is in contrast to the point multipole expansions, which generally require an infinite number of terms to exactly reproduce a given charge distribution.

Note that minimizing the error metric, Eq. (12), minimizes the error in electrostatic potential for the far-field where 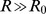, but not necessarily in the mid-field. In the mid-field (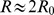), the contribution due to higher order terms in the multipole expansion can, in principle, be greater than the contribution due to lower order terms. Therefore, minimizing the lowest order terms in the expansion error does not guarantee minimization of the total error in the mid-field: these errors are investigated below for charge distributions most relevant to biomolecules. In the following analysis, for convenience, we have dropped the 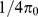 factor in Eq. (2) and (12) and switched from SI to atomic units.

### Analytical Expressions for 1- and 2-charge OPCAs

The minimization of the error metric in Eq. (12), which is required to find an OPCA, can be done numerically for any given number of 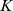 representative point charges. A numerical procedure for calculating the OPCA representation may be particularly useful in situations where the charge distributions are relatively static and thus the optimal representation does not need to be recalculated. For example, during restrained molecular dynamics simulations, components of the molecule may not move. The OPCA for these components do not need to be recalculated. For applications where the OPCA needs to be recalculated frequently, such as in unrestrained molecular dynamics simulations, one would like to find closed-form analytical expressions that can be used to compute OPCAs at a reduced computational cost and provide derivatives for force calculations.

In the following sections, we apply the general framework developed above to derive simple analytical expressions for the 1-charge OPCA. Note that the 1-charge OPCA is only applicable to a charge distribution with a non-zero net charge, since the monopole moment for a neutral charge distribution is zero. The 2-charge case is more complex: the optimal point charge approximation may result in imaginary charge values for some charge distributions (see Eq. (22) below), and can not be cast in a closed-form formula for some other distributions. Therefore, we derive more practical analytical expressions that approximate the 2-charge OPCA with a reasonable accuracy.

### 1-charge Optimal Point Charge Approximation for Charged Distributions

By definition, the 1-charge OPCA consists of a single charge. As long as the OPCA charge has magnitude 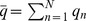, i.e. is equal to the total charge of the original distribution, the monopole term of the error expansion, Eq. (12), will be zero. Now, the remaining parameter, namely the position of the charge, is chosen to minimize the dipole term in the error expansion. In this particular case, the dipole term can be made identically zero by solving
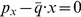
(13)

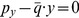
(14)

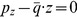
(15)for 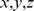 where 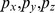 are the 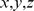 components of the dipole moment 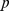 of the original distribution. Solving the above equations we have



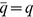
(16)

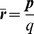
(17)


So, a charge of magnitude 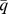 placed at 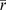 (center of charge) defines the 1-charge OPCA.

### 2-charge Optimal and Practical Point Charge Approximations

The 1-charge OPCA is the smallest set of point charges required to eliminate the monopole and the dipole term of the error expansion in Eq. (12) for systems with non-zero net charge. However, an error reduction further than the dipole order is often desired for higher accuracy. In such cases, the 2-charge OPCA (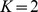) is the next step. In deriving an analytical expression for the 2-charge OPCA, the goal is to eliminate the error terms up to the dipole order in Eq. (12), and to minimize the quadrupole and, ideally, the higher order terms.

Due to important differences in the characteristics of charge distributions with zero and non-zero net charges, it is necessary to treat these two cases separately. For charged systems, the monopole and the dipole error terms are eliminated if two point charges with total charge equal to the original net charge are positioned so that their center of charge coincides with the center of charge of the original distribution. For uncharged systems, however, the monopole and dipole terms in the error expansion are eliminated when a pair of charges of equal magnitude but opposite sign are aligned with the direction of the original dipole moment. In other words, to eliminate the error terms up to the dipole in the charged case the location of the center of charge of the 2 charges is constrained, while in the uncharged case the direction of the dipole moment of the 2 charges is constrained. This leads to two different solutions for the two cases.

#### 2-charge approximation for uncharged distributions

For net zero charge distributions, the optimal point charge approximation consists of two charges 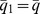 and 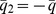 located at positions 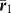 and 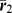 respectively. Thus, it takes 7 parameters, 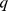, and the 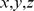 components of 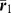, and 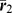, to uniquely define a 2-charge OPCA. By setting
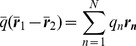
(18)the dipole term in the error is zero. Now, we will rewrite the positions 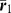 and 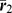 in the following form:



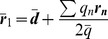


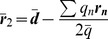
(19)where 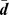 represents the geometric center between the two charges of the OPCA. We can see that these positions satisfy relation (18) automatically. By writing the positions of the charges in this manner, we have divided the process of determining the remaining parameters which define the OPCA into two steps, namely, finding the optimal placement of the charges, 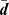, and finding the optimal magnitude of the charge, 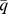. Note that finding the optimal charge value fixes the separation between the two charges, since the dipole moment of the representative distribution has been constrained to equal the original dipole moment.

**Figure 1:**
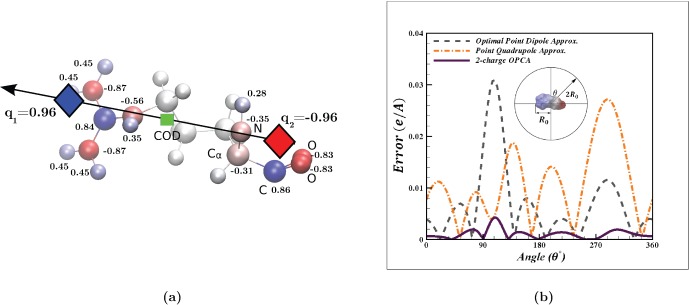
Example of a 2-charge optimal point charge approximation (OPCA). For a sample charge distribution–a neutral amino acid (C-terminal arginine at physiological pH), including the associated NH-CH-COO backbone atom. (**a**) The green square represents the center of dipole (COD), with dipole moment 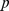 shown by the arrow. The two diamonds represent the two point charges, 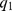 and 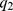, of the OPCA. The atomic partial charges are represented as spheres rendered using VMD [21]. The sphere colors range from red to blue representing the charge range of 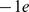 to 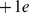, where 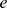 is the atomic unit of charge. The charge values for partial charges 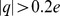 are shown next to the atoms. As a visual reference, the backbone heavy atoms are labeled and covalent bonds are included in the figure. (**b**) Error in electrostatic potential for the 2-charge OPCA, point dipole, and point quadrupole with center of dipole as the expansion center. The error is calculated relative to the exact computation, on a circle at a distance 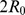, in the plane shown. Here, 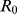 is the size of the charge distribution defined as the distance from its center of geometry to the outermost charge. The inset image shows the electrostatic surface potential rendered using GEM [22].

The placement of the geometric center 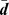 of the charges composing the 2-charge OPCA that minimizes the quadrupole term of the error expansion, is given by
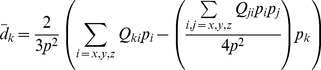
(20)where 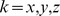, the 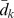
's are components of 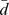, 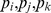 are the components of the dipole moment (Eq. (4)), and 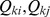 are the components of the quadrupole moment (Eq. (5)). This optimal position, known as the center of dipole, was derived previously [20] for a different purpose, namely for matching point multipole expansions between different charge distributions. Now, unlike the point dipole approximation, the 2-charge OPCA has physical size and thus an additional parameter with which to further minimize the error with respect to the given potential. In other words, Eqs. (20) and (18), determine only 6 of the 7 parameters required to define the 2-charge OPCA. Since the quadrupole moment is the lowest order non-zero term remaining in the error expansion, by choosing the optimal charge value we want to further minimize the quadrupole term in the error. However, for any charge value 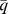 an OPCA placed at the center of dipole has no quadrupole moment as can be seen by setting 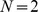, substituting the center of dipole, Eq. (20), and 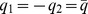 into Eq. (19), and then substituting these variables into Eq. (6). Thus, the quadrupole term in the error, Eq. (12) is unaffected by the choice of the charge magnitude 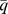, and the quadrupole term has already been globally minimized. Therefore, to uniquely define the charge 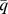, we follow the OPCA procedure and globally minimize the next term in the error expansion, namely the octupole term. Specifically, if we consider the 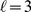 term of Eq. (12), using the connection formula from spherical multipoles to Cartesian multipoles we can compute
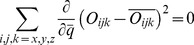
(21)where 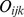 and 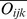 are components of the octupole moments, in Cartesian coordinates (Eq. (6)), of the original distribution and the 2-charge OPCA respectively, for an expansion computed about the center of dipole. By noting that 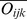 is a function of 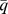, we find that Eq. (21) is satisfied when 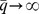 or if the charge value is given by



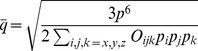
(22)Thus, Eqs. (19), (20) and (22) define the 2-charge OPCA for the net zero charge case (figure 1), i.e. defines the best placement of charges such that the error metric (Eq. (12)) is minimized.

In some cases, it is possible that
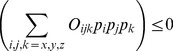
(23)


**Figure 2:**
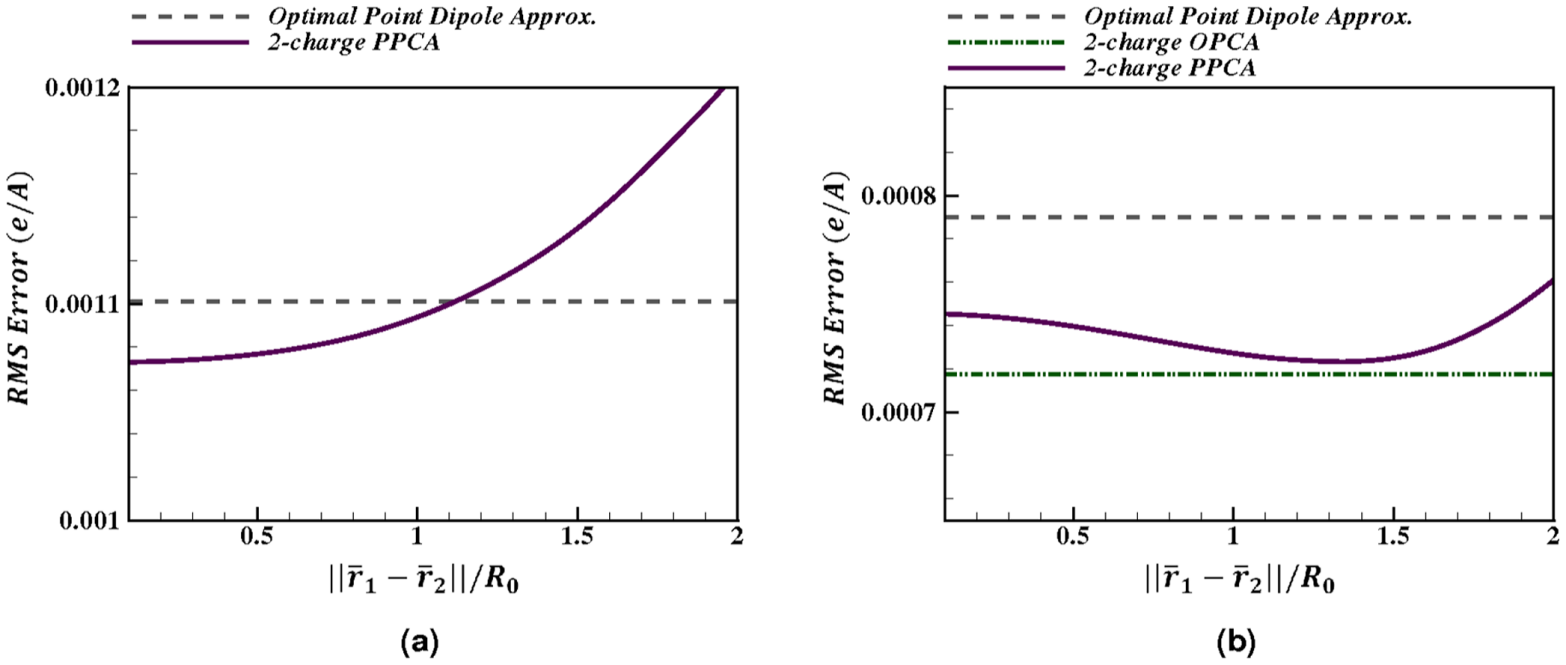
Accuracy of the 2-charge practical point charge approximation (PPCA) for charge distributions with a net zero charge. Accuracy is calculated as the RMS error relative to the exact computation, at a distance of 10 Å (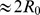) from the center of geometry. RMS error for the 2-charge PPCA (Eq. (18)) is shown as a function of the distance between the two charges of the PPCA 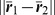. The RMS error for the 2-charge PPCA is compared to that of the point dipole approximation with an optimal center of expansion. (**a**) Cases where Eq. (23) is true. (**b**) Cases where Eq. (23) is false. This figure also includes the 2-charge optimal point charge approximation (Eq. (22)) for comparison. Connecting lines are shown to guide the eye.

In this case, the charge given by Eq. (22) is imaginary, which is unphysical. This situation occurs when the orientation of the dipole with respect to the octupole moment of the original charge distribution is such that increasing the distance between the charges of the 2-charge OPCA always increases the error. In this case, Eq. (21) is formally satisfied only for 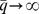. In a practical calculation, a 2-charge OPCA with inequality (23) is constructed by fixing the separation between the charges 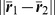 to a small value (figure 2(a)), while increasing the OPCA charge accordingly to maintain the dipole moment of the original distribution (Eq. (18)). All of the OPCA charges have real values. If inequality (23) holds, the 2-charge OPCA does not offer an accuracy advantage in the far-field over the optimal point dipole approximation, however, the 2-charge OPCA can always mimic the point dipole approximation to arbitrary precision and thus the two distributions will produce equivalent error. Thus, even if inequality (23) holds, the 2-charge OPCA represents the optimal placement of two point charges and is at least as accurate as the point dipole approximation in the far-field where 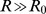. However, in the mid-field, such a charge placement may sometimes be slightly less accurate than the optimal point dipole approximation.

For the charge distributions described in the Results section below, we found that setting 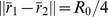 to determine the value of 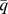 in Eq. (18), instead of the much more complex Eq. (22), results in the electrostatic potential that is on average within 4% of the optimal 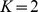 OPCA solution (figure 2(b)). Therefore, for practical applications, it may be computationally more efficient to use an empirically determined value for 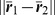 even for cases where the inequality (23) is not satisfied and a true optimal placement of 2-charge can be found via Eqs. (20) and (22). Another important practical consideration is that the contribution of each term in the error expansion, Eq. (12), is smaller than the previous term only if 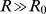. This is not necessarily true in the all-important mid-field regime where 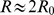. For example, for some charge distributions, the center of dipole 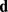 may be located at 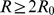. For such cases, the error for the optimal point dipole approximation [20], [23] (point dipole approximation placed at the center of dipole) and the 2-charge OPCA can become large at 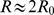. To ensure that our 2-charge approximation is reasonably accurate in the mid-field for such cases, we introduce an addition condition: the optimal charge positions are restricted to be within the 1.5 times the maximum extent of the original charge distribution 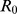, from the center of geometry.

Thus, for distributions with zero net charge, the 2-charge practical point charge approximation (PPCA), which approximates the 2-charge optimal point charge approximation (OPCA), is determined through the following 4 steps: (i) The two point charges comprising the PPCA are placed such that their center of geometry coincides with the center of dipole (Eq. (20)). (ii) The separation between the charges is fixed at 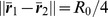. (iii) The position and magnitude of two charges are then determined by Eq. (18) and (19). (iv) In the rare cases when the point charges for the PPCA are at a distance greater than 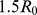 from the center of geometry, the center of dipole for the PPCA is shifted towards the center of geometry for the original charge distribution so that the point charges lie within 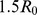. The constants in conditions (ii) and (iv) above were determined empirically for charge distributions relevant to biomolecular modeling, see the Results section below.

#### 2-charge approximation for charged distributions

The 2-charge OPCA consists of two charges 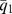 and 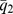. By setting
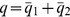
(24)where 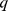 is the net charge of the original distribution, the monopole order error term in Eq. (12) becomes zero. If we set the center of charge as the center of expansion for the point dipole approximation, and choose the charges for the 2-charge OPCA such that the center of charge for the OPCA coincides with the center of charge for the original distribution, then
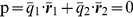
(25)where 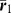 and 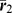 represent the position vectors for charges 1 and 2 respectively, of the 2-charge OPCA. Note that, with the choice of center of charge as the multipole center of expansion, the 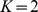 OPCA is guaranteed to be at least as accurate as the point dipole approximation, as measured by the error metric defined by Eq. (12). Thus, to simplify the derivations, we will use the center of charge as the origin for the coordinate system.

**Figure 3:**
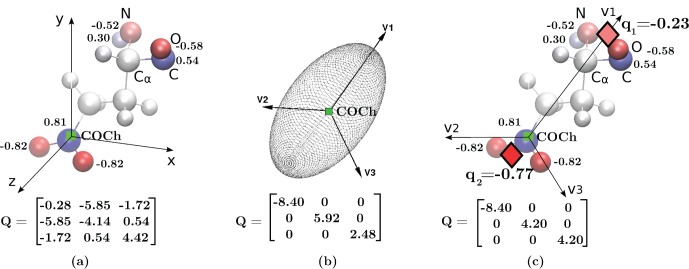
Illustration of a 2-charge practical point charge approximation (PPCA). For a sample charge distribution with non-zero net charge (a glutamic acid group within a protein with net charge 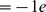, where the group includes the associated NH-CH-CO backbone atoms). (**a**) The original charge distribution with its quadrupole tensor (Eq. (5)) shown below. The atomic partial charges are represented as spheres rendered using VMD [21]. The sphere colors range from red to blue representing the charge range of 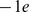 to 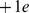. The charge values for partial charges 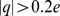 are shown next to the atoms. As a visual reference, the backbone heavy atoms are labeled and covalent bonds are included in the figure. The green square shows the center of charge (COCh). (**b**) The principal axes, **v1, v2, v3** of the original charge distribution with the center of charge as origin (green square). Its quadrupole tensor, with the coordinate system aligned to the principal axes (Eq. (27)), is shown below. Here **v1** is the principal axis with the largest principal value. Analogous to the concept of ellipsoid of inertia in Mechanics used to characterize mass distribution, an “ellipsoid of charge” can be imagined here that helps visualize the charge distribution characterized by the quadrupole tensor. (**c**) The 2-charges of the PPCA (red diamonds) are placed such that the quadrupole moment for the PPCA equals the component of the quadrupole moment for the original charge distribution along **v1**. The quadrupole tensor produced by the 2-charge PPCA, with the coordinate system aligned to the principal axes, is shown below. The values of charges are in atomic units (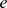), and 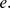
Å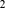 is the unit for the quadrupole tensors.

The next non-vanishing error term to be minimized is the quadrupole, i.e.
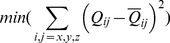
(26)where 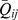 and 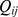 are the quadrupole moments of the OPCA and the original charge distribution respectively, about the center of charge. The quadrupole tensor defines a unique, orthogonal set of principal axes in three-dimensional space. Since the two point charges of the 2-charge OPCA define a single line, the quadrupole potential can be expected to be best approximated by the 2-charge OPCA with the charges positioned along the principal axis that has the largest absolute principal value (figure 3). Since the quadrupole tensor 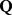 is a real symmetric matrix, its principal values can be determined by the eigenvalue decomposition
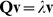
(27)where 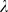 is a principal value (eigenvalue) with the corresponding principal axis (eigenvector) 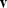. Let 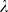 be the largest principal value. Then, by placing the 2-charge OPCA along 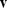, and setting the component of quadrupole moment for the 2-charge OPCA along the principal axis 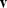 equal to the largest principal value 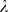 for the original distribution, we obtain from Eq. (5) for the quadrupole moment
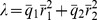
(28)where 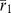 and 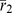 are the magnitude of the 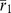 and 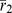 vectors with center of charge as the origin.

Substituting the values for 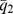 and 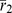 from Eq. (24) and (25) respectively, we arrive at:
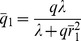
(29)


**Figure 4:**
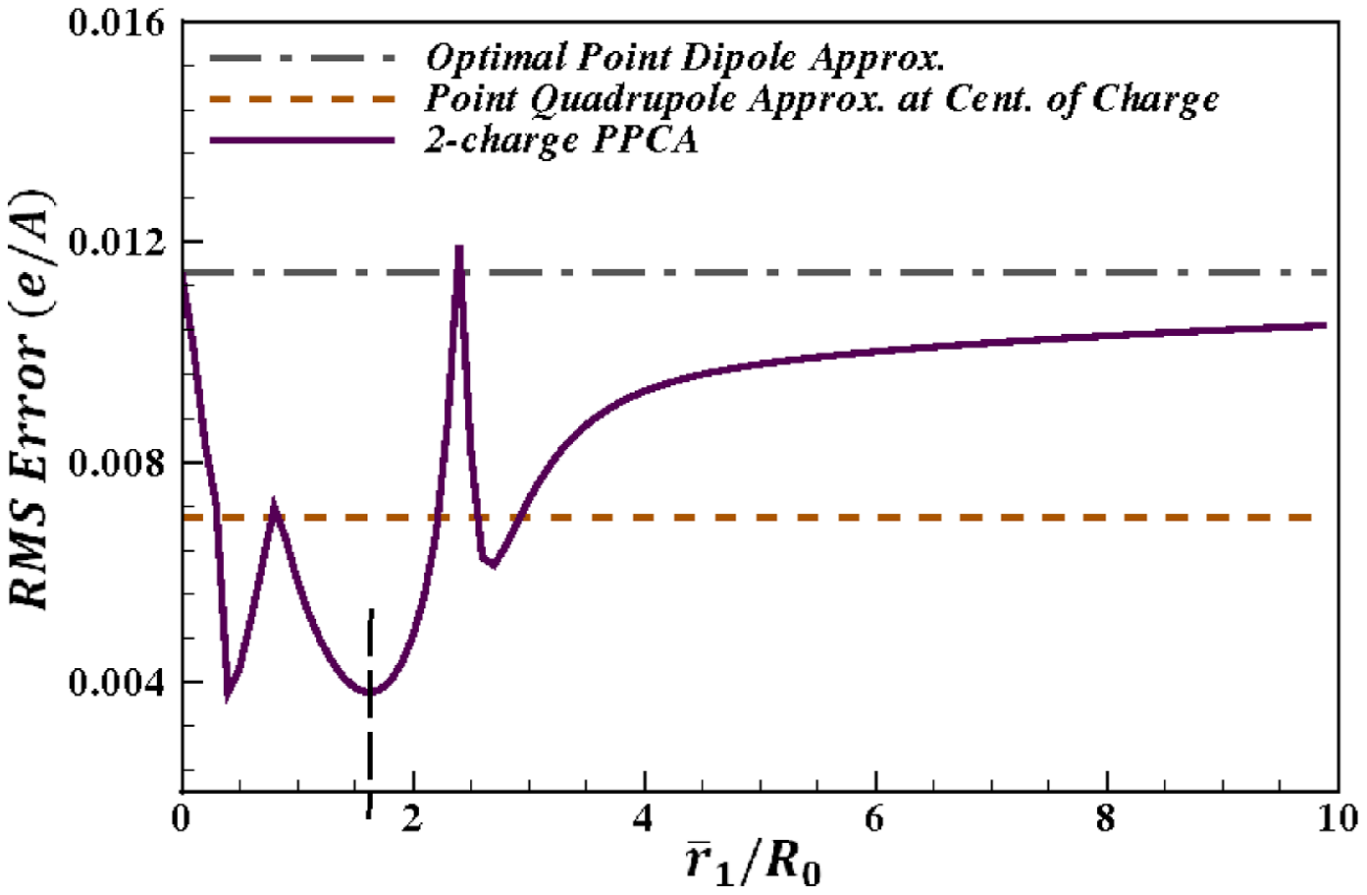
Accuracy of the 2-charge practical point charge approximation (PPCA) as a function of the distance 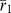 from the center of charge. For the sample charge distribution shown in figure 3. Accuracy is calculated as the RMS error, relative to the exact computation, at a distance of 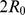, where 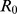 is the maximum extent of the charge distribution from the center of geometry. The point dipole and point quadrupole approximations with center of charge as the center of expansion are shown for comparison. The vertical dashed line represents the value 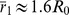 that produces the lowest RMS error for the 2-charge PPCA in this case. Connecting lines are shown to guide the eye.

The above equation does not provide a unique solution since 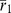 is still unknown. Minimizing the error in the next order multipole term in Eq. (12), the octupole moment, results in quartic equations which may produce imaginary charge values. Therefore, for practical applications, as with the uncharged case, an empirical approximation may be more appropriate. Specifically, we set 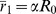 where 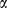 is an empirical parameter. Consider for example a typical charge distribution (a glutamic acid) from the sample charge distributions described in the Results section below. For this charge distribution, figure 4 shows that in the mid field (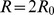), with the choice of 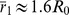 this practical point charge approximation (PPCA) is on average more accurate than the point dipole and point quadrupole approximations. For the representative sample charge distributions described in the next section, the PPCA was found to be the most accurate for 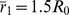.

By placing the 2-charge PPCA along the principal axis with the largest principal value, we eliminate the error due to the largest component of the quadrupole tensor 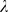. Furthermore, since the quadrupole tensor is traceless, the other two principal values 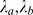 in 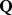 and 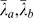 in 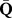, are of the opposite sign to 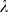, and 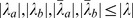. Therefore, the error due to the other two components of the quadrupole tensor are reduced as well, i.e. 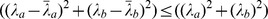. As an illustration, consider the example of figure 3(b): the error due to smaller components of the quadrupole tensor shown in figure 3(b) are smaller than the ones in figure 3(c), as 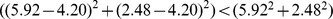.

Thus, for a charge distribution with net non-zero charge, the practical point charge approximation is determined by Eq. (29), with 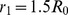. The constant of 1.5 was empirically determined for the set of sample charge distributions described in the following section.

## Results and Discussion

We consider here two potential applications for the optimal and practical point charge approximations developed above – the approximation of atomic partial charge distributions for amino acid groups within proteins, and the approximation of the charge distribution of water molecule.

### Atomic Level Biomolecular Modeling

Molecular modeling is commonly used to study the structure, function and activity of biological systems [24]–[26]. A common computational bottleneck in biomolecular modeling is the calculation of long-range electrostatic interactions: due to slow decay of these interactions with distance, simply ignoring them beyond a certain cut-off distance may lead to unacceptable accuracy loss [16], [27]. Multiscale approximations are one class of methods used to speed up these calculations [5], [7], [16], where near-field interactions are treated exactly, while an approximation of the charge distribution is used for mid- and far-field computations.

Since the error introduced by such approximations is generally very low in the far-field, understanding the mid-field error of such approximations, including ours, is most relevant. In the context of biomolecular modeling, we consider the lower bound of the mid-field to be no less than 2 times the extent of the charge distribution (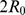); the mid-field for amino acid groups is therefore greater than 5 Å.

We have applied the 2-charge practical point charge approximations (PPCA) developed above to the computation of electrostatic potential for a set of 1188 amino acid groups in five representative biomolecules that span a large range of sizes: a monomer from the virus capsid (Protein Databank (PDB) ID 1A6C) with 513 groups, the villin headpiece protein (PDB ID 1VII) with 36 groups, calcium switch protein (PDB ID 1UWO) with 91 groups, chaperonin GroEL (PDB ID 2EU1) with 524 groups, and myoglobin (PDB ID 1YMB) with 24 groups. The amino acid groups include their associated backbone atoms, NH-CH-CO for non-terminal groups, NH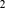-CH-CO for N-terminal groups, and NH-CH-COO for C-terminal groups. Atomic partial charges were taken from the AMBER force field parameters [28]. The electrostatic potential was calculated in the mid-field (for two values: 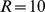 Å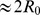 and 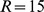 Å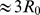) where the approximation is likely to be least accurate. The electrostatic potential was computed at discrete points on a sphere of radius 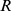, centered at the center of geometry. The spherical surface was discretized into 7200 grid points at which the electrostatic potential was calculated. The RMS error was calculated over all grid points and all the amino acid groups in the sample as
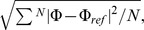
 where 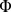 and 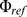 are the electrostatic potential calculated using the approximations and the reference (original) charge distributions, respectively, and 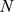 is the number of grid points. The 2-charge PPCA was compared to the optimal point dipole and the point quadrupole approximations. The center of expansion for the point dipole approximation for uncharged and charged distributions are chosen to be the center of dipole and center of charge, respectively, which are known to be optimal [20] for the corresponding point multipole expansions. For the point quadrupole approximation, we found that the choice of center of geometry as the center of expansions for uncharged cases, and the center of charge for charged cases, produced the most accurate result, on average, in the mid-field. Accordingly, we use these points as the expansion centers for the point quadrupole approximation.

**Figure 5:**
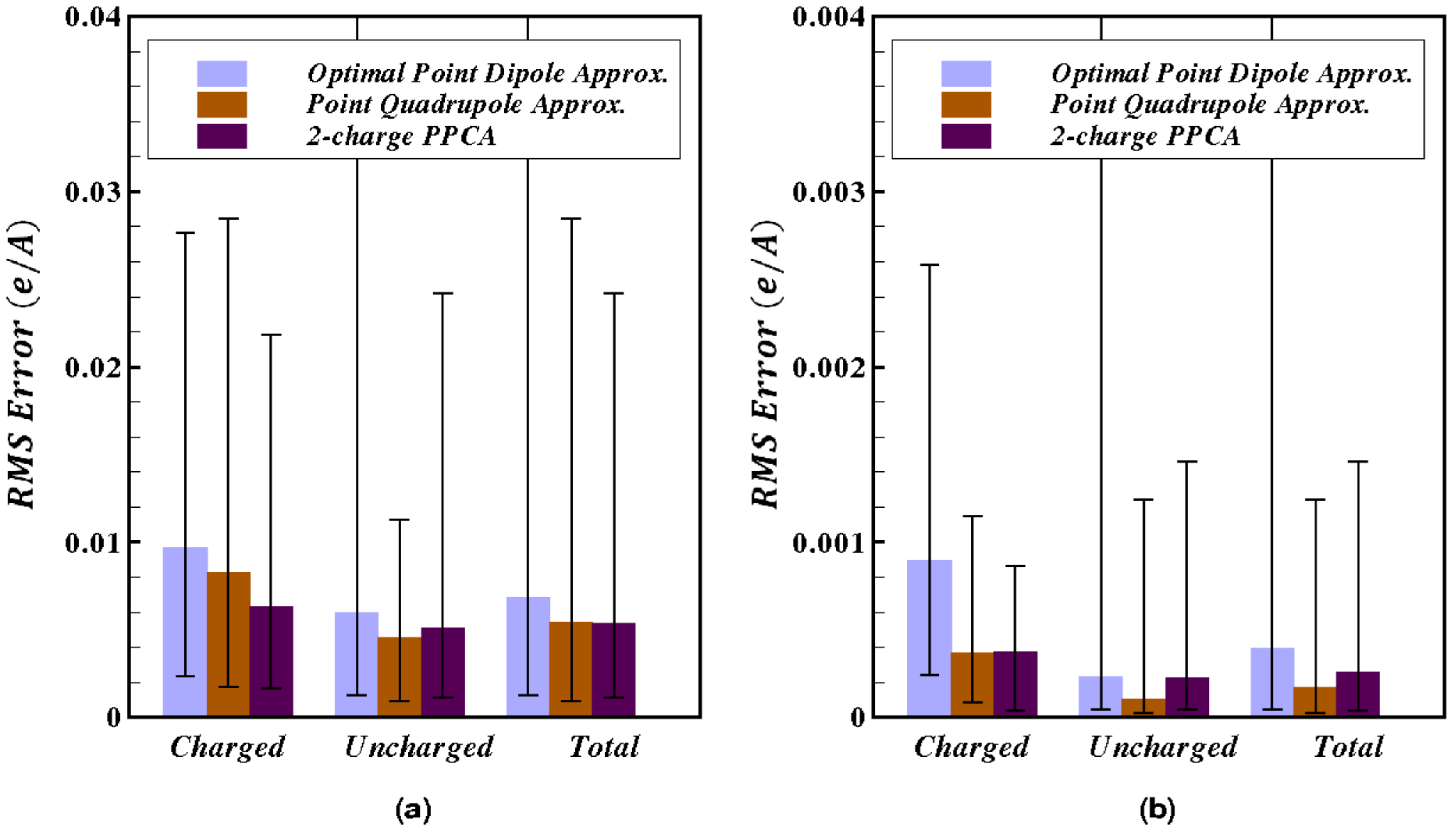
Accuracy of the 2-charge practical point charge approximation (PPCA). For a sample set of charge distributions relevant to biomolecular modeling. Accuracy of the point dipole and point quadrupole approximations are shown for comparison. Accuracy is calculated as the RMS error relative to the exact computation. (**a**) Error calculated at a distance of 10 Å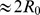 where 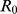 is the maximum extent of the charge distribution from the center of geometry. (**b**) Error calculated at a distance of 15 Å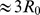 from the center of geometry. Error bars show the maximum and minimum absolute error. The upper values for the error bars that are cut off at the top are 0.14 and 0.006 in the left and right panels, respectively.

In the mid-field (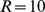 Å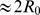) the RMS error (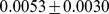 e/Å) for the 2-charge PPCA is comparable to the point quadrupole approximation RMS error (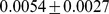 e/Å), and 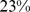 less than the optimal point dipole approximation RMS error (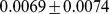 e/Å), for the charge distributions considered here, figure 5. On the other hand, when electrostatic potential is calculated at a distance 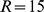 Å, the RMS error (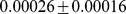 e/Å) for the 2-charge PPCA is 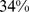 less than the optimal point dipole approximation RMS error (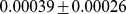 e/Å), while being 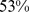 higher than the point quadrupole approximation RMS error (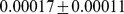 e/Å). These results reflect the fact that the 2-charge PPCA is always at least as accurate as the optimal point dipole approximation, whereas the PPCA can only try to minimize the error in the quadrupole term unlike the point quadrupole approximation, which eliminates the error in the quadrupole term. As the distance from the charge distribution increases, the accuracy of the multipole expansion, and, specifically, the accuracy of the point quadrupole approximation improves. This is evident from the errors at a distance 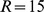 Å(figure 5(b)) which are an order of magnitude lower compared to the errors at a distance 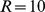 Å (figure 5(a)). Note that the set of amino acid groups used here consist of approximately 20% charged and 80% uncharged distributions.

Figure 5(a) also shows that the 2-charge PPCA is on average significantly more accurate than the point dipole and quadrupole approximations for net non-zero charge distributions, compared to net zero charge distributions. Thus, the 2-charge PPCA should be significantly more accurate than the point dipole and quadrupole approximations for molecular structures that contain a significant portion of charged amino acids. And this is precisely the type of structures where the use of long-range cut-offs may lead to large errors. Also note that the standard deviation in RMS error for the 2-charge PPCA (0.0030 e/Å) is comparable to that of the point quadrupole approximation (0.0027 e/Å) and is less than half of that of the optimal point dipole approximation (0.0074 e/Å). Thus, the 2-charge PPCA is a considerably “tighter” approximation than the equivalent order optimal point dipole approximation. The higher standard deviation in RMS error for the point dipole approximation is primarily due to the cases where the center of dipole, for charge distributions with zero net charge, falls outside the extent of the original charge distribution. In these cases the “optimal” center of expansion for the point dipole approximation can be very close to the point at which the electrostatic potential is approximated, resulting in large errors. This source of error is explicitly removed from the PPCA.

### Optimal Point Charge Approximation for Water Molecule

Water is critical for life [29], [30], and is one of the most extensively studied molecules[31]–[34]. Accurate yet computationally efficient description of the solvent environment is essential for realistic biomolecular modeling. Commonly used simple fixed point charge models of water have achieved a reasonable compromise between accuracy and speed, but these are by no means perfect [35], [36]; the search for more accurate yet computationally facile models continues [4], [37], [38]. The ability of a given model to reproduce electrostatic properties of the highly polar water molecule is critical to success of the model [4]. Obviously, any reasonable model needs to account for the large dipole moment of water molecule in order to reproduce dielectric properties of the liquid state. But higher moments are important too: for example, one of the components of the water quadrupole tensor is large, and was shown to have strong effect on the liquid water structure seen in simulations [39]. The octupole order terms have also been shown to be of importance: for example, these affect water structure around ions [40]. An intricate interplay between the dipole, quadrupole and octupole moments gives rise [41] to the experimentally observed charge hydration asymmetry of aqueous solvation – strong dependence of hydration free energy on the sign of the solute charge. Thus, accurate yet computationally facile representations of the complex charge distribution of water molecule should be of interest.

**Figure 6:**
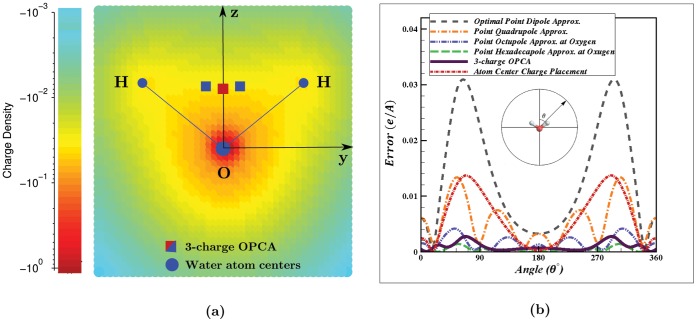
3-charge optimal point charge approximation (OPCA) for water molecule. (**a**) The quantum mechanical electron charge density is visualized by a light blue to red colormap representing the charge density range of 0 to 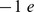 per 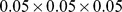 Å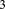. The figure shows a 3 Å 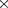 3 Å slice of the charge distribution in the y-z plane of the water atom centers. The origin is located at the center of the oxygen atom, the water atoms lay in the y-z plane, and the z-axis bisects the hydrogen atoms. The blue dots represent the water atom centers and the red and blue squares represent the 3 OPCA charges. The central OPCA charge has a value of 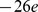 and the other two are 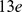 each. (**b**) The error in electrostatic potential relative to the exact computation, calculated at 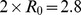 Å from the oxygen atom, in the y-z plane. In this case 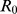 is chosen to be 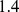 Å, the mean van der Waals radius of water [42], and 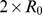 approximates the distance between the oxygen atoms in two closest water molecules. For comparison, we show the error for the 4 lowest point multipole approximations as well as for a commonly used approximation which places point charges on atom centers. To match the dipole moment of the original charge distribution, the charge placed at the oxygen position equals 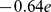, while the charges on the hydrogen centers are 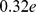 each. The same relative ordering of errors is seen in the x-z and x-y planes (not shown).

As an illustration of the OPCA approach, we show here that the 3-charge OPCA can accurately reproduce a quantum mechanical charge distribution of the water molecule up to the octupole moment. The specific charge density for the electron distribution of the water molecule used here (Fig. 6(a)) was determined by the CCSD method with aug-cc-pCVTZ basis set [43]–[45] at experimental equilibrium geometry in the gas phase. The electron charge density distribution was calculated for a box with side length of 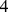 Å and resolution of 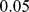 Å. The resulting multipole moments of water molecule in the gas phase are comparable to available experimental values [46] (Table 1). We stress, however, that the specific charge distribution is used here only to illustrate the OPCA method and its capabilities; in what follows, no claims regarding suitability of this distribution for simulation of liquid phase water [39] are made.

**Table 1:** Multipole moments of a water molecule in the gas phase.

		QM (this work)	3-charge OPCA	Experimental
Dipole(*D*)	*p_z_*	1.81	1.81	1.86
Quadrupole(*DÅ*)	*Q_zz_*	0.08	0.08	0.11
	*Q_xx_*	−2.53	−2.53	−2.625
	*Q_yy_*	2.45	2.45	2.515
Octupole(*DÅ* ^2^)	*O_zzz_*	−1.35	−1.17	NA
	*O_xxz_*	−1.25	−1.44	NA
	*O_yyz_*	2.61	2.61	NA

The computed values for the quantum mechanical (QM) charge distribution and the 3-charge OPCA are compared to the corresponding experimental values [46]. The coordinate system is that of Figure 6(a). Due to the symmetry, for the octupole tensor 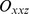 = 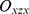 = 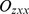 and 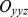 = 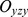 = 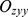. Components of multipole moments with a value of zero are not shown. 1 debye (D) = 0.2082 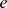
Å.

Since the water molecule is neutral, it can not be represented by a 1-charge OPCA. A 2-charge OPCA can accurately represent the dipole moment but not the quadrupole and octupole moments of the distribution, which are important for an accurate representation of water [36], [39]–[41]. Therefore, we calculate the 3-charge OPCA, as follows. In general, the 3-charge OPCA consists of three charges 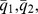 and 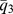, located at 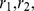 and 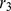, representing 12 independent variables. But in the case of water any solution must respect the 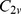 symmetry of the molecule, which reduces the number of independent variables to 10 (by assuming 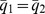 and 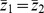). Following the general procedure outlined in the “Calculating the optimal point charge approximation” section above, we first eliminate the monopole term in the error expansion Eq. (12), by setting
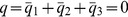
(30)where 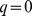 is the monopole moment of the original charge distribution for water. Then, we eliminate the dipole term in the error expansion via
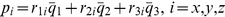
(31)where 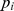 are the 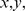 or 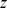 component of the dipole moment and 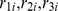 are the 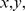 or 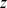 components of 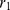, 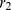 and 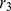. Note that in the coordinate system standard for water molecules (figure 6(a)), 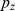 is the only non-zero component of the dipole moment. Finally, we eliminate the quadrupole term in the error expansion, Eq. (12), by setting
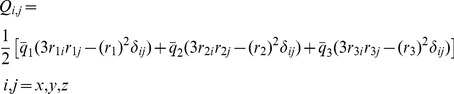
(32)where 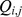 are the terms in the quadrupole tensor of the original charge distribution. Note that in the coordinate system chosen (figure 6(a)), all off-diagonal terms in the quadrupole tensor are zero, i.e. 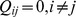. Thus, we are left with a total of 9 independent equations –1 for the monopole 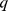, 3 for the dipole terms 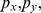 and 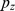, and 5 for the terms in the symmetric traceless quadrupole tensor, 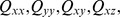 and 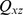 – to solve for 10 variables. This is an under-determined system of equations, leaving one additional variable. Solving the above set of equations results in the following solution, with 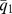 as the remaining variable.



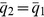
(33)

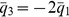
(34)

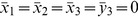
(35)

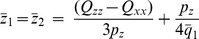
(36)

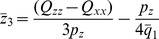
(37)

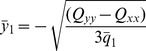
(38)

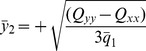
(39)


The value for 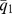 is determined numerically to minimize the octupole term in the error expansion, Eq. (12). The resulting OPCA charges are 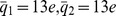, and 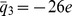, located at (0, −0.16, 0.49), (0, 0.16, 0.49), and (0, 0, 0.47) Å, respectively, (Fig. 6). (The conversion factor of 0.2082 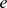
Å/debye is used to convert the multipole moments in table 1 to atomic units (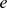
Å, 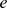
Å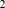, etc.) used in Eq. (33) – (39).)By construction, the multipole moments from the 3-charge OPCA and the quantum mechanical charge distribution are identical up to quadrupole order, as shown in (Table 1). The tight clustering of the 3 OPCA charges away from the oxygen nucleus is unexpected – in many commonly used water models the charges are placed on atom centers. Mathematically, the clustering results from minimizing the error at the octupole level, used to determine the value of 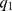 in Eq. (33)–(39). For fixed dipole and quadrupole moments, the extent of the OPCA charge distribution is controlled by the octupole moment of the original charge distribution. The small distance between the opposite OPCA charges necessitates their large magnitude to ensure correct dipole moment of the charge distribution. Although the position and magnitude of the OPCA charges may appear unusual, the OPCA representation may be more accurate than the atom-centered alternative. For example, when we place the point charges on atom centers, and adjust the corresponding charge magnitudes so that the error at the dipole order is eliminated, the RMS error in electrostatic potential at 2.8 Å around the oxygen atom center is 0.0073 e/Å, which is many times larger than the RMS error for the 3-charge OPCA (0.0010 e/Å), as shown in Fig. 6(b). Note that the OPCA representation is designed to best approximate multipole moments of the original charge distribution; it remains to be seen whether this strategy leads to accurate reproduction of other physical properties of water. A water model based on the OPCA representation will be presented in a separate study.

Figure 6(b) compares the error in electrostatic potential calculated by the 3-charge OPCA with the error produced by the point multipole approximations, relative to the exact computation using the original charge distribution. (Error calculations exclude any points that fall within the extent of the original charge distribution.) The error shown in figure 6(b) is calculated on a circle, in the plane of the water atoms (y-z plane), at 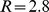 Å from the oxygen atom, which approximates the oxygen-oxygen (contact) distance between two closest water molecules. The overall RMS error in electrostatic potential calculated on a 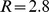 Å spherical surface centered at the oxygen atom is 0.0010 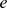/Å with maximum error of 0.0027 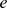/Å for the 3-charge OPCA, compared to 0.0015 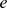/Å with maximum error of 0.0041 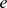/Å for the point octupole expansion. The overall RMS error in electrostatic potential at 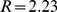 Å (experimental oxygen-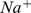 distance [47] ) is 0.0036 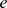/Å (1.20 kcal/mol) with maximum error of 0.0042 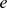/Å (1.39 kcal/mol) for the 3-charge OPCA compared to 0.0065 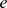/Å (2.16 kcal/mol) with maximum error of 0.0074 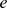/Å (2.46 kcal/mol) for the point octupole expansion.

## Conclusions

Truncated point multipole expansions are a widely used approach to approximate potentials produced by complex charge distributions. However, if only the lowest order terms in the multipole expansion are kept, as is often done in practical calculations, the point multipole expansion can produce considerable error in the mid-field. Furthermore, implementation of such approximations into existing electrostatic models that were originally developed for point charge distributions, *e.g.* pairwise implicit solvent models, presents many challenges. In this work, we have introduced an alternative to the point multipole expansion–the optimal point charge approximation (OPCA). An OPCA consists of a given number of point charges which are optimally placed to best reproduce the electrostatic potential due to the original charge distribution. By construction, OPCAs retain many of the useful properties of point multipole expansions, in particular they retain the asymptotic behavior of the point multipole expansion. At the same time, an expansion based on OPCAs can be more accurate than the point multipole expansion of the same order.

We have provided a general framework for calculating OPCAs to any order. We have also derived closed-form analytical expressions for the 1-charge OPCA, and closed-form analytical expressions that approximate the 2-charge OPCA with reasonable accuracy – the 2-charge practical point charge approximation (PPCA). We note that higher order closed-form, analytical OPCAs may be challenging to derive, but for some applications, lower order OPCAs may be sufficient. The analytical expressions derived here for the 1-charge and 2-charge OPCAs, are guaranteed to be at least as accurate as the corresponding point multipole expansion of the same order. These analytic expressions not only provide physical insight but are more computationally efficient than the numerical minimization procedures that are in general required to obtain the optimal point charge approximation. Thus, these analytic expressions may be particularly useful in applications such as molecular dynamics where computational speed is critical.

For a set of sample charge distributions relevant to biomolecular modeling, the 2-charge PPCA was found to be on average 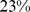 more accurate than the point dipole approximation, and comparable in accuracy to the point quadrupole approximation in the mid-field (electrostatic potential evaluated at 2 times the extent of the charge distribution). The standard deviation in RMS error for the 2-charge PPCA was also 59% lower than that of the point dipole approximation and comparable to that of the point quadrupole approximation, suggesting that the approximation offered by the PPCA is “tighter” than that of the point dipole.

We also calculated the 3-charge optimal point charge approximation to represent a (quantum mechanical) gas phase charge distribution of water molecule. The electrostatic potential approximated by the 3-charge OPCA in the mid-field (2.8 Å from the oxygen atom) is on average 33.3% more accurate than that of the point octupole approximation. Interestingly, the positions of the 3 OPCA charges are quite different from atom center charge placements based on simple point charge models such as SPC or TIP3P. Further investigation is necessary to determine if and how such a 3-charge approximation can be used in practical applications.

Representing complex charge distributions by a small number of point charges is not, by itself, a novel idea. There are a number of methods, such as RESP [48], CHELP [49], CHELPG [50], CHELMO [19], Finite Point Charge (FPC) [3], coarse graining [16], [51], [52] and others [53] that empirically fit a set of point charges to a given charge distribution by minimizing various error metrics in electrostatic potential over some volume or surface surrounding the charge distribution. However, a key difference between the above methods and the optimal point charge approximation introduced here, is that the OPCAs (and their practical approximations, PPCAs) inherit the physically appealing asymptotic properties of the point multipole approximation, i.e. the error in potential is guaranteed to fall off at least as fast as 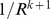, where 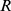 is the distance from the origin and 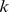 is the highest order of the multipole terms retained in the expansion. In contrast, fitting the representative charges to minimize electrostatic error over some arbitrary volume or surface (e.g. molecular surface) does not guarantee the above asymptotic behavior, and can potentially lead to relatively large errors outside the volume or surface used for fitting.

Furthermore, in comparison to point multipoles, expansions based on PPCAs have many desirable properties that may be useful in practical computations; in particular, their mathematical form – the sum of Coulombic contributions from point sources – is simpler than that of the conventional point multipole expansion and is amenable to common speed-up schemes such as the generalized Born implicit solvent model [54]. Thus, PPCAs may be easier to implement into existing molecular dynamics protocols.

The optimal point charge approximation presented here is a new concept; thus its many applications and potentially useful properties remain unexplored in this proof-of-concept work. We expect OPCA/PPCA to have utility in coarse-grained [51], [55] and multi-scale methods [16], especially in dynamics [27] where analytic expressions and the simplicity of the algorithms is key. The approximations we have introduced provide a systematic way of deriving approximate charge distributions that have the potential to be both computationally efficient and produce an accurate representation of the original electrostatic potential. To further improve the representation of the original potential via OPCAs, future work may consider partitioning the original charge distribution into several domains, and finding OPCA/PPCA for each of them separately, similar to the distributed multipoles approach [56]–[58]. Further exploration of the mathematical and physical properties of OPCAs is also desirable. Finally, the 3-charge OPCA for a charge distribution representing water molecule is quite accurate to the octupole order. This accuracy, combined with the simplicity of a 3-charge OPCA, is noteworthy.
